# Challenges and Insights in Absolute Quantification of Recombinant Therapeutic Antibodies by Mass Spectrometry: An Introductory Review

**DOI:** 10.3390/antib14010003

**Published:** 2025-01-07

**Authors:** Sarah Döring, Michael G. Weller, Yvonne Reinders, Zoltán Konthur, Carsten Jaeger

**Affiliations:** 1 Federal Institute of Material Testing and Research (BAM), 12489 Berlin, Germany; sarah.doering@bam.de (S.D.); michael.weller@bam.de (M.G.W.); zoltan.konthur@bam.de (Z.K.); 2 Leibniz-Institut für Analytische Wissenschaften—ISAS—e.V., 44139 Dortmund, Germany; yvonne.reinders@isas.de

**Keywords:** monoclonal antibody, therapeutic antibodies, mass spectrometry, liquid chromatography, absolute quantification, isotope labeling, metrology, traceability, certified reference material

## Abstract

This review describes mass spectrometry (MS)-based approaches for the absolute quantification of therapeutic monoclonal antibodies (mAbs), focusing on technical challenges in sample treatment and calibration. Therapeutic mAbs are crucial for treating cancer and inflammatory, infectious, and autoimmune diseases. We trace their development from hybridoma technology and the first murine mAbs in 1975 to today’s chimeric and fully human mAbs. With increasing commercial relevance, the absolute quantification of mAbs, traceable to an international standard system of units (SI units), has attracted attention from science, industry, and national metrology institutes (NMIs). Quantification of proteotypic peptides after enzymatic digestion using high-performance liquid chromatography-tandem mass spectrometry (HPLC-MS/MS) has emerged as the most viable strategy, though methods targeting intact mAbs are still being explored. We review peptide-based quantification, focusing on critical experimental steps like denaturation, reduction, alkylation, choice of digestion enzyme, and selection of signature peptides. Challenges in amino acid analysis (AAA) for quantifying pure mAbs and peptide calibrators, along with software tools for targeted MS data analysis, are also discussed. Short explanations within each chapter provide newcomers with an overview of the field’s challenges. We conclude that, despite recent progress, further efforts are needed to overcome the many technical hurdles along the quantification workflow and discuss the prospects of developing standardized protocols and certified reference materials (CRMs) for this goal. We also suggest future applications of newer technologies for absolute mAb quantification.

## 1. Historical Overview: Evolution of Recombinant mAbs

The development of hybridoma technology by Köhler and Milstein in 1975 provided the cornerstone for the generation of monoclonal antibodies. By fusing B lymphocytes from immunized animals with myeloma cells, stable cell lines for unlimited monoclonal antibody (mAb) production could be obtained for the first time [1]. Through their ability to bind with high selectivity and affinity to a wide range of targeting biomolecules, mAbs gained increasing attention for application in life science and medicine. The first murine mAb approved by the Food and Drug Administration (FDA) for therapeutic application in humans was Muromonab in 1986 [2]. Unfortunately, the murine nature of antibodies generated by hybridoma technology bore some disadvantages for their human therapeutic application. The immune system recognized the injected mAbs as foreign proteins, resulting in the production of antibodies against those, the so-called human anti-mouse antibody (HAMA) response [3]. Consequently, the administered antibodies were eliminated from the body, reducing the effectiveness of treatment or causing allergic reactions [4].

**Figure 1:**
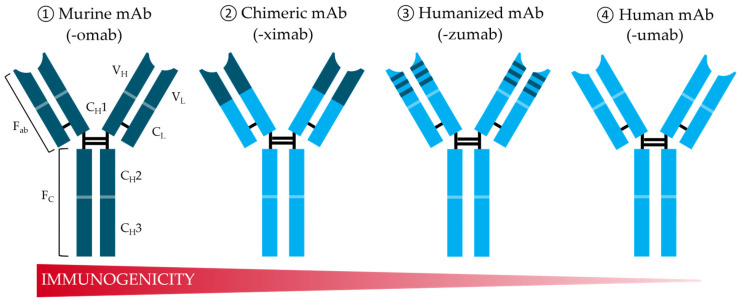
Schematic overview of therapeutic mAb structure and its evolution from murine antibodies (dark blue domains) to fully human antibodies (light blue domains) with an associated decrease in immunogenicity in humans. The constant domains of the heavy chain (C_H_2 and C_H_3) are localized in the fragment crystallizable (Fc) region. The antigen-binding F_ab_-fragments consist of the constant (C_L_) and variable (V_L_) parts of the light chains as well as the variable (V_H_) and constant domain (C_H_1) of the heavy chain. Adapted from [17].

To overcome such limitations, the field of recombinant DNA technologies offers a wide variety of tools for structural modifications of murine mAbs to create more human-like antibodies (Figure 1) and enable large-scale production using mammalian cell lines. Initial achievements have been made by combining the variable light (V_L_) and heavy (V_H_) domains of murine mAb ① with a constant domain of human light chain (V_C_) and F_C_-region (fragment crystallizable) to generate chimeric antibodies ② with reduced but still observable immunogenicity in humans [5,6]. The first FDA-approved chimeric antibodies were Abciximab in 1994 [7] and Rituximab in 1997, a recombinant antibody produced in Chinese hamster ovary (CHO) cells [8]. Further developments enabled the reduction of murine parts in mAbs, such as complementarity-determining region (CDR) grafting, whereby it was possible to incorporate hypervariable loops from V_L_ and V_H_ of the antigen-binding sites (F_ab_) of the murine antibody into a fully human antibody scaffold [9] (Figure 1, ③). These humanized antibodies were even less immunogenic than chimeric mAbs but, in some cases, showed reduced affinity to their target antigen [10]. The first FDA-approved humanized mAb was Daclizumab in 2016, an immunosuppressive agent used to reduce renal transplant rejection in patients [11]. Since then, various methods have been developed for the humanization of therapeutic antibodies to improve their properties and make them applicable to medical treatment [12].

Another notable step in the evolution of therapeutic antibodies was the development of antibody phage display technology [13], based on the previously established phage display method by Smith et al. in 1985 [14]. For this approach, a library of antibody gene fragments from immunized or non-immunized animals as well as humans is needed. Additionally, fully synthetic libraries were established, as reviewed recently by Zhang et al., 2023 [15]. The expressed antibody fragments are presented as fusion proteins with the coat protein of M13 bacteriophage surfaces and allow selection against the targeted antigen. In contrast to traditional hybridoma technology, this in vitro selection method enables the creation of mAbs even against toxic and non-immunogenic agents. The impact of phage display on the development of therapeutic antibodies has been reviewed in detail by Frenzel et al., 2016 [16].

Further inventions in the field of structural modifications of mAbs were made by Lonberg et al. in 1994. By replacing the entire murine immunoglobulin repertoire in the mouse genome with those of humans, it was possible to create fully human antibodies [18]. Such transgenic mice produce human-like antibodies after immunization, followed by conventional hybridoma technology to obtain fully human monoclonal antibodies (Figure 1, ④). Panitumumab, the first fully human mAb isolated from a transgenic XenoMouse, was FDA-approved for therapeutic application in 2006 [19]. However, this approach is limited in cases where the immunogens are toxic or show a high degree of homology between the targeted human antigen and its murine ortholog.

Despite the mentioned limitations, the creation of chimeric, humanized or fully human antibodies was a breakthrough and led to a wave of FDA-approved antibodies of Iimmunoglobulin G antibody class 1 (IgG1) and 4 (IgG4). Many of them belong to the best-selling pharmaceuticals worldwide, like the blockbuster Adalimumab, which has effectiveness against rheumatoid arthritis and other chronic or immune-inflammatory diseases [20,21] (Table 1). Other relevant antibodies, such as Pembrolizumab and Nivolumab, are used in targeted melanoma therapy [22]. Among those common anti-cancer antibodies, modified antibody-drug conjugates (ADCs) were developed for targeting cancer antigens and delivering small, chemically linked cytotoxic agents to tumor cells. Currently, only thirteen FDA-approved ADCs for numerous cancer variants are on the market [23]. Some major limitations during clinical trials were their modification heterogeneity due to non-specific drug conjugation and dynamic in vivo change after application [24]. Therefore, it is not surprising that many quantitative methods exist for the investigation of pharmacokinetic properties of ADCs in human plasma or serum [25,26,27]. With increasing market importance, analytical methods for the quantification of conventional therapeutic mAbs are also gaining relevance in clinical and pharmaceutical analysis. These methods are crucial for ensuring product quality, safety, and efficacy, particularly as mAbs are used in life-saving therapies such as cancer treatment. Nevertheless, the complex nature of mAb structures presents distinctive challenges, and highly sensitive and specific methods are essential for the precise quantification of these biomolecules.

## 2. Current Strategies in MS-Based Quantification of Antibodies

Over the last years, high-performance liquid chromatography coupled with tandem mass spectrometry (HPLC-MS/MS) became further established as one of the main technologies in antibody quantification [38]. Many innovative HPLC-MS/MS-based approaches were reported, focusing on more selective, more sensitive, faster and/or simplified detection of different antibody species in human blood or serum. Most of the methods continued to quantify antibodies on a peptide level; however, studies directly measuring intact or partially digested antibodies were also increasingly presented. These methods are mainly used for the quantification of antibodies with potential matrix effects, for example, in the clinical context of pharmacokinetic parameters such as antibody metabolism (half-life) or distribution in the patient’s body [39]. Moreover, several authors reported advancements in absolute amino acids analysis for the quantification of purified antibodies. In addition to the methods mentioned above, this strategy is particularly suitable for quality control of pharmaceutical antibody formulations in industry or accurate protein quantification of reference material products. Consequently, these reference materials can be employed to develop novel MS-based protein quantification techniques. Furthermore, they are metrologically traceable, thereby enhancing the comparability and reliability of measurement outcomes on a global scale.

**Figure 2:**
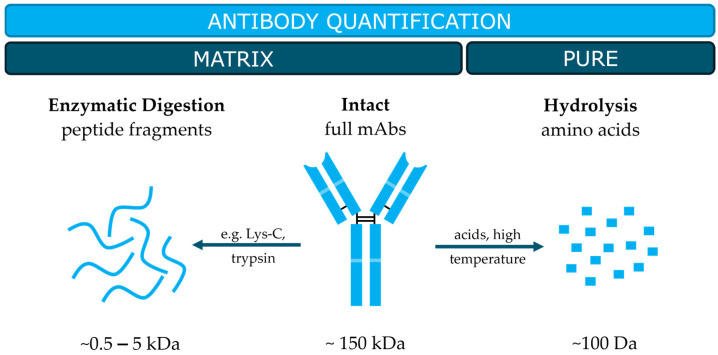
Overview of MS-based antibody quantification strategies of pure or matrix-containing antibody samples. Proteolytic enzymes cleave the antibody sequence at specific positions, such as the C-terminal bond of lysine and arginine (trypsin), releasing defined peptide fragments. The treatment of antibodies with acids under high temperatures enables quantification of free amino acids.

An overview of the mentioned quantification strategies and target applications is provided in Figure 2. Developments in peptide-based methods will be discussed in detail in the next section; “intact” strategies and amino acid-based quantification will be shortly outlined after that. The selected literature was based on reviewing the methods used in the quantification of commercially important antibodies, as listed in Table 1, followed by a systematic review of primary publications that highlight problems in antibody and protein quantification from different perspectives.

### 2.1. Quantification of Enzymatically Digested Antibodies

One key advantage of peptide-based quantification is the high sensitivity and selectivity that can be achieved for these analytes on commonly available triple-quadrupole (QqQ) mass spectrometers. Typically, a complex biological sample such as human blood serum containing the target antibody (or antibodies) is enzymatically digested into numerous defined peptides of varying chain lengths. The peptide mixture is chromatographically separated and contains one or more abundant peptides identified as unique to the antibody of interest. These so-called “signature peptides” originate only from the antibody sequence and are used for further MS/MS analysis. For quantification, the signals of these signature peptides are related to those of spiked analog standards in known amounts, preferably represented by stable isotope-labeled (SIL) signature peptides (Section 3.2) or co-digested signature peptides of intact SIL antibody analogs (Section 3.1). By comparing the signal from the endogenous signature peptides with the SIL peptides, the exact amount of the target antibody in the sample can be determined with high precision.

To date, no universally applicable protocol exists for peptide-based mAb quantification. Instead, individual method development and validation remain necessary for each mAb, as different sample characteristics affect digestion efficiency, peptide recovery, signal-to-noise ratios, and so on [40]. This section reviews steps in sample preparation and peptide selection frequently reported as critical for robust quantification of mAbs in biological samples.

#### 2.1.1. Purification and Enrichment

Due to interfering matrix components (proteins, lipids, DNA), HPLC-MS/MS analysis of antibodies in human serum usually requires an enrichment step in sample preparation. This can be carried out before or after the digestion step. Enrichment prior to digestion concentrates the mAb fraction and often uses magnetic beads or solid-phase extraction (SPE) cartridges, either coupled with anti-idiotypic antibodies for CDR-specific purification [41] or protein A and G that specifically bind to the constant Fc-region of mAbs [31,36,37,42]. Precipitation with organic solvents (e.g., methanol) or inorganic salts (ammonium sulfate) is an inexpensive and simple method for isolating the overall protein fraction prior to MS analysis [43]. Enrichment of targeted peptides after digestion is very commonly performed using C18-reversed phase/cation exchange SPE cartridges or tips, a step that also removes residual salts that may form excessive adducts during electrospray ionization (ESI).

It was also noted that each sample processing step carries the risk of analyte losses. For example, a significant sample loss of approx. 40% and poor quantitative precision of ±15% was observed by Heudi et al. from SPE-based peptide cleanup of digested mAb [38]. This highlights the need to optimize SPE conditions individually for each assay and to include internal standards right from the beginning of the experiment.

#### 2.1.2. Denaturation, Reduction, and Alkylation

Most published protocols include denaturation and reduction steps prior to digestion to unfold the tertiary structure and break intramolecular disulfide bonds of proteins. This facilitates access of the enzyme to the cleavage sites, ensuring efficient digestion. For the chemical denaturation of proteins, the use of buffers containing strong chaotropic denaturants such as urea or guanidine hydrochloride [44,45] in final concentrations > 6 M and ionic detergents like sodium dodecyl sulfate (SDS) and sodium deoxycholate (DOC) [44,45] was reported. Proc et al. demonstrated that the use of guanidine hydrochloride consistently yielded lower digestion efficiency than urea, SDS or DOC-based denaturing protocols [46]. Additionally, guanidine hydrochloride can inhibit trypsin activity even at low concentrations, rendering a buffer exchange step necessary. High urea concentrations can also influence digestion efficiency, but sample dilution to a urea concentration < 1 M was sufficient for complete trypsin digestion [38]. As urea is heat-sensitive, it can degrade in different buffers into ammonium cyanate, which binds to free amines by carbamylation reactions, resulting in structure modifications of proteins [47]. However, if SDS remained in the buffer solution during trypsin digestion, the proteolytic enzyme denatured and subsequent MS analysis was affected by ionization suppression at levels as low as 0.01% [48,49].

Several MS-compatible detergents were made commercially available, such as ProteaseMax (Promega GmbH, Walldorf, BW, GER), RapiGest™ surfactant (Waters Corporation, Milford, MA, USA), PPS Silent Surfactant (Agilent Technologies, Santa Clara, CA, USA) as well as Progenta (Protea Biosciences, Morgantown, MV, USA), which degrade proteins in combination with heat or in the low pH range [50]. For example, Abe et al. diluted a Nivolumab-containing sample with RapiGest™ surfactant and denatured it by heating it above 80 °C [36]. Also, organic solvents were used at specific concentrations for enrichment by precipitation as well as sample denaturation [30] without a noticeable reduction in enzyme activity [46]. Each of the mentioned methods has its drawbacks and needs to be optimized experimentally.

The reduction step during denaturation is essential for allowing the proteolytic enzyme to access the entire protein structure. Dithiothreitol (DTT) is commonly used as a reduction agent [30,36,38,45,51]. As an alternative, tris(2-carboxyethyl)phosphine (TCEP) was used [52,53]. TCEP had higher pH as well as temperature stability and faster-reducing properties as compared to DTT. In the context of bottom-up sequencing, it was recently shown that the use of different reducing agents had only a minor impact on peptide identification [54]. By contrast, the influence of different alkylation agents on peptide yield was apparently larger. Alkylation agents derivatize free thiol groups of cysteines, thereby inhibiting their ability to reform disulfide bonds. The most frequently used alkylation agent for antibody quantification is iodoacetamide (IAM) [36,38,51,53]. The reaction is carried out in the absence of light and at room temperature to avoid side reactions. Over the past few years, different alkylation reactions have been investigated in detail. Mueller and Winter compared IAM as an alkylation agent against iodoacetic acid (IAA), acrylamide (AA) and chloroacetamide (CAM) [55]. The authors identified unspecific reactions on the side chains of tyrosine, serine and threonine of digested HeLa proteins when using the iodine-containing reagents. Contrary to this, another study reported a higher yield of alkylated cysteine peptides and fewer side reactions when the procedure was performed using IAA compared to AA, N-ethylmaleimide or 4-vinylpyridine [54]. However, investigations by Kuznetsova et al. revealed that carbamidomethylation may affect up to 80% of peptides containing methionine after IAM-alkylation of digested proteins from HeLa and HepG2 cells [56]. Robinson and Hains have found that less light-sensitive chloroacetamide has an adverse impact on methionine oxidation [57]. Furthermore, IAM increased the rate of methionine-to-isothreonine conversion; structurally related IAA induced the same side reactions. Additionally, it has been demonstrated that IAM-alkylated peptides with N-terminal cysteines are prone to cyclization, which can result in a mass shift of −17 Da [58]. Consequently, optimization of reduction and alkylation conditions appears fundamental for antibody quantification. Due to the time-intensive optimization procedure as well as possible by-products, there are many protocols that skip these steps [28,29,30,35,37]. However, this can lead to incomplete protein digestion, as the antibody only partially unfolds or even refolds, and disulfide-connected peptides complicate the software-supported evaluation [59].

#### 2.1.3. Digestion

Efficient enzymatic breakdown of therapeutic mAbs into peptides is crucial for reliable determination of the antibody amount by this approach. Specifically, digestion efficiencies appear to depend on the individual protein structure, in particular regarding phosphorylation and glycosylation patterns [59,60,61]. Proteases should be of the highest “MS grade” quality to ensure high specificity, activity, and purity. For MS analysis, peptides with basic residues at the C-terminus are preferred due to their enhanced ionization efficiency in positive electrospray ionization (ESI+) mode. Therefore, it is not surprising that the serine protease trypsin is the most frequently used proteolytic enzyme. Several trypsin products from different suppliers are available on the market (Table 2), varying in activity and optimal digestion conditions. It should be noted that naturally obtained trypsin from porcine or bovine pancreas can contain impurities of chymotrypsin. To prevent the protease activity of chymotrypsin, the tosyl-phenylalanyl-chloromethyl-ketone (TPCK) is added to most commercial trypsin products. Furthermore, autolysis of trypsin results in the formation of pseudotrypsin with a chymotrypsin-like activity. Both drawbacks can be avoided by using recombinant and modified trypsin, whose dimethylated lysine residues prevent self-digestion and increase stability [62,63]. Surface-immobilized trypsin is increasingly being implemented for antibody digestion because it offers faster digestion with less self-digestion [36,64] and can be integrated as immobilized enzyme reactors for automated sample processing [65]. Nevertheless, non-oriented immobilization may also result in a reduction in the accessibility of the enzyme’s active site [66] and higher quantification limits in comparison to in-solution digestions [65].

**Figure 3:**
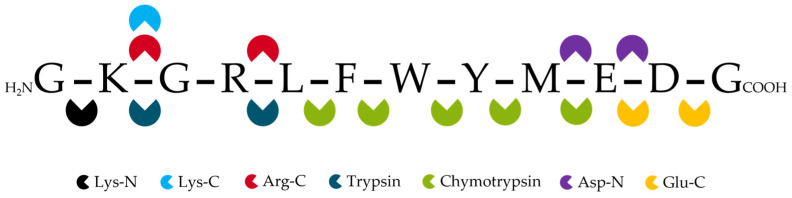
Cleavage sites of commonly used proteases for bottom-up proteomics.

Trypsin cleaves proteins specifically at the C-terminus of the basic amino acids arginine (R) and lysine (K) (Figure 3), typically generating peptides of a size favorable for downstream HPLC–MS/MS analysis. Both amino acids are abundant and well-distributed in different proteins, producing peptides with an average length of 14 amino acids in length and a minimum of two positive charges [59]. However, when a proline residue follows at the C-terminus of R or K, the protein backbone is almost unaffected by trypsin. Furthermore, the presence of glutamic and aspartic acid, as well as phosphorylated threonine and serine, may increase the frequency of so-called missed cleavages. Therefore, some providers offer protease products combining standard trypsin with the digestion of Lys-C (Table 2), which shares C-terminal K as the cleavage site but continues to work under high urea concentrations (6–8 M urea). The combination minimizes missed K cleavages, resulting in increased digestion efficiency and improved reproducibility [67].

Sometimes, trypsin fails to generate peptides of optimal length due to the high abundance of R and K in the antibody sequence. In such cases, the use of other proteases like chymotrypsin, AspN, GluC, and ArgC can be good alternatives [68,69]. Here, the nomenclature of these enzymes corresponds to the target amino acid and terminus at which the protein is cleaved (Figure 3). Generally, enzymatic digestion should be performed in a buffer with a compatible pH that is optimal for the specific enzyme. For example, the listed products in Table 2 recommend the use of ammonium bicarbonate or Tris buffers with a pH between 7 and 8. However, Tris enhances the formation of various adduct ions and can cause ion suppression during ESI [49]. The selection of an appropriate buffer can also influence the generated peptides, whereby spontaneous chemical deamination of asparagine and glutamine residues may occur [70,71].

Furthermore, enzymatic proteolysis can be performed under different temperatures, digestion times and enzyme-to-antibody ratios. Typically, digestion with trypsin is carried out at 37 °C for one to 24 h (Table 2). Some optimized or immobilized trypsin variants are stable at higher temperatures and enable faster digestion [59]. Additionally, the incubation time can depend on the position of target signature peptides and their steric accessibility. Peptides, especially from CDRs located at the surface, are easily accessible and allow fast enzymatic digestion [72]. Lastly, prolonged digestion time and elevated temperatures can introduce unwanted protein modifications and amino acid changes like asparagine deamidation and N-terminal glutamine cyclization [70,71,73], as well as oxidation [74]. For enzymatic digestion, an enzyme-to-antibody ratio of 1:100 to 1:20 is commonly recommended. Nevertheless, it is mandatory to check digestion specificity and completeness for absolute quantification.

#### 2.1.4. Signature Peptide Selection

Quantification of mAbs on a peptide level requires the selection of suitable signature peptides that allow both selective and sensitive detection of the target antibody in the sample matrix (Table 1). The signature peptide should consist of a unique amino acid sequence for the desired antibody and have good chromatographic separability and ionization efficiency for HPLC-MS/MS analysis. Knowledge of the amino acid sequence of the target antibody is advantageous for this selection. Generally, protein sequences of FDA-approved mAbs are available in the IMGT^®^ (ImmunoGenetics Information system, http://www.imgt.org/ (accessed on 7 August 2024)) or databases of ABCD (AntiBodies Chemically Defined, https://web.expasy.org/abcd/ (accessed on 7 August 2024)). For the prediction of potentially generated peptides, the free programs MS-DIGEST from Protein Prospector (http://prospector.ucsf.edu (accessed on 7 August 2024)) or the deep learning program DeepDigest [75] can be used. When quantifying antibodies in human serum, peptides of the CDR are preferable due to their uniqueness for each mAb as well as enzymatic accessibility. Accordingly, predicted peptide sequences can be analyzed with the Basic Local Alignment Search Tool (BLAST, https://blast.ncbi.nlm.nih.gov/Blast.cgi (accessed on 7 August 2024)) for proteins or Peptide Atlas (http://www.peptideatlas.org (accessed on 7 August 2024)) for matches with interfering peptide sequences of the human proteome. For the quantification of purified therapeutic antibodies, choosing peptides from the constant Fc-region can also be used, allowing the quantification of multiple mAbs of similar subclass with the same MS assay.

Nevertheless, the selection of suitable signature peptides for quantitative measurements can be affected by some analytical and equipment limitations. Initially, the selected peptides should have a length of 8 to 25 amino acids. Often, shorter sequences are not unique, while longer sequences usually lead to a significant charge distribution [69]. In addition, the amino acids contained in the peptide candidates should be considered based on their susceptibility to chemical modifications and instability that have become known in recent years [76,77]. For example, cysteine, methionine, tryptophan, and histidine are well-known amino acids prone to oxidation and leading to a mass shift of +15.9949 Da. Furthermore, asparagine or glutamine can undergo deamidation during trypsin digestion, causing mass shifts of −17.0265 Da and +0.9840 Da, respectively. The loss of ammonia through N-terminal glutamine cyclization induced by prolonged digestion time also leads to a loss of −17.0265 Da. It should also be noted that therapeutic antibodies can undergo oxidation or deamination during production, purification or storage and may, therefore, already be present before enzymatic digestion [76]. Especially for pharmacokinetic quantification studies of antibodies, biotransformation effects like blood oxidation, deamidation or proteolytic degradation should also be considered [39]. In this context, signature peptides that are rich in potentially modifiable amino acids should be avoided. Other important tips for selecting the right signal peptides for targeted protein quantification have been discussed elsewhere [78,79,80].

Likewise, peptides potentially carrying glycan or phosphorylation residues should be treated with care. Falck et al. have shown that the conserved glycopeptide EEQYNSTYR of IgG1 antibody contained a high mannose content or was present as a sialylated or more complex glycoform, and a statistically significant trypsin cleavage rate was observed. This peptide should, therefore, be excluded from analysis due to unreliable quantification. [60]. Software tools such as NetNglyc [81] (https://services.healthtech.dtu.dk/services/NetNGlyc-1.0/ (accessed on 7 August 2024)), NGlyc [82] (https://github.com/bioinformaticsML/Ngly (accessed on 7 August 2024)), or the newer deep neural network-based approach DeepNGlyPred [83] (https://github.com/dukkakc/DeepNGlyPred (accessed on 7 August 2024)) can be used to check peptide sequences for potential glycosylation issues. Moreover, Solari et al. mentioned ionization and digestion issues with phosphopeptides for protein quantification in their study and recommended utilizing higher trypsin-substrate ratios or dephosphorylation prior to HPLC-MS/MS analysis to address these challenges [61].

### 2.2. Quantification of Intact Antibodies

In contrast to peptide-based quantification, this strategy enables the direct measurement of intact antibodies without digestion and minimal sample preparation. Due to the high molecular weight of antibodies, the quantification occurs using high-resolution mass spectrometry (HRMS) or quadrupole mass analyzers. For quantification, the antibody sample is spiked with an intact SIL analog standard (Section 3.1) in a known amount. By comparing the peak signals of the target antibody to the SIL standard, the exact concentration of the intact antibody in the sample can be determined.

This approach has proven particularly useful in pharmacokinetic studies to monitor the biotransformation of therapeutic mAbs in patients. Jian et al. published a method for absolute quantification of intact mAbs in plasma, using an isotope-labeled mAb as an internal standard and automated software-assisted mass peak deconvolution [84]. Selective preconcentration and sample clean-up of targeted antibodies from the interfering matrix were achieved by immunoaffinity capture (IAC). Similarly, a ligand binding assay (LBA) was integrated into a high-resolution (HR)-MS workflow for simultaneous quantification of different human IgG1s at intact level, including an isotopic-labeled variant as internal standard [85]. The authors validated the method regarding selectivity, sensitivity, accuracy and precision, carryover, dilution linearity as well as reproducibility and found similar performance compared to peptide-based quantification. In addition, deglycosylation of intact human IgG1 with the enzyme PNGase F resulted in a less complex full-scan MS spectrum and increased the signal for each charge state. Notably, the use of PNGase F also allows the parallel quantification of N-glycan structures, which are involved in many biological processes. Methodological concepts for this purpose have been discussed elsewhere [86,87].

However, absolute quantification of intact mAbs by HPLC-MS/MS involves a range of drawbacks. The main problem relates to the high molecular weight of antibodies [88]. Additionally, the molecular heterogeneity due to post-translational modifications (PTMs) and chemical modifications, especially O-/N-glycosylation but also phosphorylation, deamidation and oxidation, increases the complexity of the analysis [89]. Compared to peptide-level analysis, the combination and varying location of such modifications results in more and partially interfering analyte signals that need to be monitored. As the complexity of charge state and isotope distributions increases with mass in ESI mass spectra, signal intensity decreases, resulting in a loss of analytical sensitivity. Although deconvoluted spectra can circumvent this problem to some degree [90,91], their use for quantification purposes is currently not recommended due to potential issues in data processing [88]. Furthermore, the necessary enrichment of mAbs with IAC prior to HPLC-MS/MS analysis adds additional complexity and has been critically reviewed by Zhao et al. [92] If IAC enrichment is unavoidable and the SIL internal standard is used for quantification, its addition to the matrix prior to sample preparation is compulsory [93]. For quantification of intact mAbs, the commercially available isotope labeled human IgG SILu™ mAb has been applied in a few studies [84,94]. Furthermore, the mAbXmise kit from Promise Proteomics (Table 2) also contains SIL-mAbs for frequently occurring antibodies in the field of inflammatory and oncological therapies, e.g., Adalimumab [32,95]. Similarly, in-house production of SIL analogs of targeted mAbs was also investigated [85].

### 2.3. Quantification of Hydrolysed Antibodies

Amino acid analysis (AAA) refers to a broadly used, absolute quantification method for targeting amino acids hydrolytically released from proteins and peptide samples. Commonly, hydrolysis of 1 to 10 nmol of protein is carried out under acidic as well as oxygen-free conditions using 6 M hydrochloric acid (HCl), elevated temperatures between 90 to 150 °C and incubation times ranging from 1 to 72 h [96,97,98]. To reduce reaction time, the application of microwave-assisted heating is widely used [98]. However, substances like salts, metal ions, nutrients or hydroxyl radicals in the hydrolysis solution can potentially affect the release of certain amino acids [96,99,100]. For example, iron, copper, but also fat can lower tyrosine recoveries but can be removed by an additional SPE cleaning step. Phenol, by contrast, can prevent amino acid degradation and halogenation of tyrosine during the hydrolysis process [96,98].

The preferred HPLC modes used for AAA of hydrolyzed proteins are hydrophilic interaction chromatography (HILIC), ion exchange (IEX) or reversed phase (RP) chromatography [99]. Conventionally, AAA included a derivatization step to optimize chromatographic separation as well as UV sensitivity for UV/VIS detection. With advances in HPLC-MS/MS analysis, accurate quantification of underivatized amino acids has now become widespread. The omission of derivatization is beneficial, as it eliminates the risk of potential contamination that may arise during the derivatization process [101].

Analogous to the absolute quantification of peptides, HPLC-MS/MS allows absolute AAA by including SIL internal standards in the analysis. Here, SIL internal standards can be full-length protein analogs [102] (Section 3.1), peptides (Section 3.2) or amino acids [103,104] (Section 3.3). By comparing the peak signals of the natural amino acids to those of the SIL standards, the exact concentration of selected amino acids from the antibody can be determined. When certified reference materials (CRMs) are used, measurements become fully traceable to the international standard system of units (SI units) [98]. This system ensures consistency and standardization in measurements around the world, making it easier for people to share and compare data. By using four SIL amino acids as internal standards in an HPLC-MS/MS setup, Jeong et al. developed an accurate quantification method for human growth hormone with an intra- and inter-day precision of relative standard deviation (RSD) of less than 1% [105]. Accordingly, the absolute quantification of proteins by AAA allows traceable and comparable results, as well as the certification of therapeutic protein products with certified SIL reference materials. Interlaboratory comparisons for therapeutic antibody quantification [106] have proven the validity of this technique. Furthermore, this approach has also contributed to the development of several protein reference materials, such as COVID-19 analytical reagents [103,104,107].

## 3. Selection of Internal Standards for Quantification with HPLC-MS/MS

**Figure 4:**
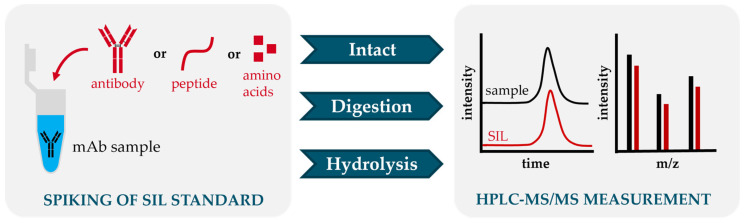
Schematic principle of SIL internal standard integration for the different quantification methods of mAbs. The sample is spiked with known concentration of SIL internal standard antibody, peptide, or amino acids (red). Due to the chemical similarity, both sample and SIL standard elute at the same time and differ only in their mass-to-charge (*m*/*z*) ratios.

The choice of suitable internal standards is critical for quantitative MS, as losses during digestion and hydrolysis, as well as matrix effects, can lead to significant technical bias in quantification. SIL internal standards contain at least one stable isotope (usually ^13^C, ^15^N or D) in at least one amino acid, which usually ensures co-elution with the respective target peptide or amino acid. Due to the known concentration of the added SIL internal standard, the unknown concentration of antibodies in the sample can be determined based on the signals in the HPLC-MS/MS analysis (Figure 4). Accurate quantification of mAbs was demonstrated using both SIL proteins and peptides, which in turn can be quantified by means of SIL peptides or amino acid standards. To enable metrological comparability and reliability of measurement results, calibration should be performed using SIL internal standards that can be traced back to reference materials.

### 3.1. Intact Antibody Standards

Different SIL antibody standards were presented for mAb quantification at the intact level. Mainly, commercially available SIL-mAbs were used and offered from different suppliers like Promise Proteomics [108] or Sigma Aldrich. The use of full-length [32,37] or partial [32,37] SIL analogs for digested mAbs were also tested and included in quantification protocols. These internal standards can be calibrated using reference materials of known quantity, the so-called protein standard absolute quantification (PSAQ) method [102,109]. For example, Lebert et al. demonstrated simultaneous quantification of several therapeutically relevant and structurally similar IgG1 and IgG4 isotypes using PSAQ [52]. For in-house production of SIL mAbs, the antibody-producing cell line is allowed to grow in media containing isotopologues of one or more amino acids, which are incorporated into the desired mAb [110]. Although SIL amino acids are readily available, the approach is usually cost- and time-intensive [93].

A more cost-effective alternative for peptide-based quantification with antibody standards is to use SIL mAbs that produce identical or similar peptides in enzymatic digestion, allowing multiple therapeutic antibodies to be quantified simultaneously. For instance, the quantification of the therapeutic antibodies infliximab, alemtuzumab, and bevacizumab using SIL mAb analogs showed sufficient precision, i.e., coefficient of variation (CV) of less than 20%, when signature peptides had a similar amino acid sequence and were located in the V_H_- and V_L_-region [111]. Furthermore, the purchasable human IgG1 SILuMAb with SIL arginine and lysine residues can also be a less expensive alternative for peptide-based quantification due to a common F_C_ region with other therapeutic mAbs. However, as noted by Smit and co-workers, the digestion kinetics can vary between SIL mAbs and the mAbs of interest when different recombinant expression systems were used, likely due to associated variations in post-translational modifications [112].

In summary, internal standardization with SIL mAbs can provide crucial advantages in terms of reproducibility and robustness. Due to the identical behavior of targeted and SIL antibodies, throughout the entire analytical workflow, variability and inferences in MS analysis can be reduced to a minimum. However, a significant drawback is the overall cost and quality of SIL antibody standards, which currently restricts their broader application.

### 3.2. Peptide Standards

SIL peptide standards generally share the same AA sequence with the selected signature peptides. SIL peptides can be easily synthesized in HPLC-grade purity and are thus much cheaper than intact SIL mAbs. SIL peptide-based mAb quantification has become common practice over the last decade [36,42,51,53]. An important factor is the concentration and purity of SIL peptides that should be verified by AAA to obtain precise reference values [98,113]. Burkitt et al. established a method for protein quantification in which the SIL peptide standards were quantified by isotope dilution MS (IDMS) [114]. By using certified amino acid reference materials, mAb quantification was fully traceable to SI units.

In contrast to antibody standards, SIL peptides can only be corrected for variability in instrumental factors and matrix effects but not for changes in sample purification or enrichment prior to digestion. Extended SIL peptides contain additional amino acids adjacent to the cleavage site of the signature peptide. They can improve tracing digestion efficiency as additional amino acids need to be eliminated during enzymatic cleavage. Benesova et al. demonstrated that SIL peptides extended by three amino acids at each N- and C-terminus enable quantification results for albumin equivalent to those based on a SIL protein [115]. However, digestion kinetics can also differ for targeted mAbs and extended SIL peptides due to the accessibility of the cleavage site. For example, Li et al. obtained higher precision with a SIL intact antibody (<16% CV) than with SIL peptides in both standard and extended variants (>25%) [116]. Therefore, it is often recommended to measure at least two signature peptides to compare cleavage rates and to produce more robust results [42,117].

Peptide modifications occurring during sample preparation are another factor that can prevent accurate quantification of mAbs. For instance, CDR-specific SIL peptide standards prone to methionine oxidation impaired the quantification of therapeutic mAbs [74]. Similarly, the quantification of growth hormone indicated that the addition of the SIL peptide prior to digestion was crucial for correcting peptide degradation rates [117].

Because IgG1 and IgG4 are the primary subclasses used for therapeutic antibodies (Table 1), SIL peptides of either the constant heavy or constant light chain have the advantage of being applicable for quantification of multiple therapeutic mAbs [118,119].

### 3.3. Amino Acid Standards

As previously described, only a subset of the 20 proteinogenic amino acids have proven to be sterically accessible and generally stable enough for the acidic hydrolysis procedure. For example, peptide bonds between aliphatic/hydrophobic amino acids are difficult to break, resulting in insufficient release and underestimation without additional hydrolysis time. Furthermore, the chemical oxidation of methionine or deamination of asparagine and glutamine into their acid counterparts makes these amino acids unsuitable for quantification. Although correction factors were developed, the extent of hydrolysis losses and degradation artifacts were shown to vary between laboratories. Therefore, absolute protein quantification by AAA is currently limited to specific amino acids, with proline, valine, isoleucine, leucine, and phenylalanine being favored in the literature [103,104,106,107,114].

## 4. Software Tools Supporting Targeted mAb Quantification

The commonly used acquisition mode for absolute mAb quantification at the peptide or amino acid level is multiple reaction monitoring (MRM), using highly sensitive and selective QqQ or quadrupole-ion trap (Q-Trap) mass spectrometers. Although targeted peptides or amino acids and their spiked SIL analogs exhibit nearly identical physicochemical properties (e.g., retention time and fragmentation patterns), the MS1 monoisotopic mass shifts depending on the incorporation of labeled isotopes. Quantification based on MRM involves extracted ion chromatograms (XICs) that are created for both the precursor and product *m/z* of the selected peptides or amino acids. By relating peak areas of added SIL standard to those of the target peptides or amino acids, the antibody concentration can be precisely determined. The development of efficient MRM schemes depends on many critical factors such as chromatographic separation, selectivity of transitions, as well as fine-tuned fragmentation energies. Furthermore, metrological parameters, including a limit of detection and quantification (LOD, LOQ), accuracy, precision, linearity, and stability, following FDA [120] or European Medicines Agency (EMA) [121] guidelines, must be taken into consideration during method development. Necessary procedures for the latter have already been discussed in detail for beginners elsewhere [79,122]. Here, we focus on commercial and open-source software packages that support method development and data analysis but can themselves pose a challenge for inexperienced users in the field (Table 3). Software tools enabling time-efficient and user-friendly processing of large sample numbers obtained from different HPLC-MS/MS setups are also discussed. Furthermore, solutions for the automated prediction of tryptic peptides and their organization into spectral libraries are addressed.

### 4.1. Commercial and Device-Specific Software

All leading MS manufacturers offer workflow-oriented software solutions supporting mAb characterization, partially in combination with pre-configured instrument stacks, such as the BioAccord System (Waters Corporation, Milford, MA, USA). The latter is specifically designed for routine and automated monitoring of biotherapeutics in quality control processes, including multi-attribute method (MAM) workflows for analyzing antibody degradation and modification, e.g., during storage [123] and quantification of mAbs at the intact level [124]. More general software for MRM analysis by the same manufacturer includes the TargetLynx™ Application Manager (method development, automated sample data acquisition, processing, and reporting) and QuanOptimize™ (automated optimization of MRM transitions and collision energies). Similarly, BioPharma Finder^TM^ (Thermo Fisher Scientific, Waltham, MA, USA) is a comprehensive software package offering pre-defined workflows, e.g., enabling quantification at the intact level due to meticulous deconvolution of mass spectra or multi-attribute characterization of mAbs in contexts such as bioprocessing up to final product quality control [35,125,126]. To identify proteotypic peptides and predict fragmentation patterns for targeted quantification, Pinpoint^TM^ (Thermo Fisher Scientific) helps to determine MRM transitions and can export methods directly to compatible instruments. Due to an iterative workflow design, acquired data can be used to refine acquisition methods and verify top peptide candidates [127,128]. MassHunter (Agilent Technologies, Santa Clara, CA, USA) includes various packages for data acquisition and quantitative analysis. MassHunter Optimizer supports the development of MRM methods by automatically optimizing acquisition parameters, facilitating, e.g., the development of more sensitive triggered MRM (tMRM) methods [129]. The Analyst^®^ software suite (version 1.7.3, AB Sciex, Framingham, MA, USA) not only provides general MS data acquisition and analysis but also offers a variety of tools specifically designed for the analysis of mAbs. These tools include intact mass spectrum characterization, subunit analysis, peptide mapping, glycan analysis, and drug-antibody ratio (DAR) calculations (Biologics Explorer, ProteinPilot^TM^). In addition, MRMPilot^TM^ supports building and optimizing peptide MRM experiments, allowing inspection of full scan MS/MS data as well as transferring transition settings to the acquisition module. MRMPilot^TM^ also allows input from open databases such as PeptideAtlas [130]. Similarly to MassHunter, it also supports the developing of MRM-triggered acquisition methods. For data evaluation, MultiQuant^TM^ (AB Sciex) supports absolute quantification based on multiple peptides and the creation of reports including common quality parameters such as the CV and accuracy.

Furthermore, third-party software primarily aimed at high-throughput proteomics can be applied to targeted quantification tasks. Available options include Spectronaut^TM^, Spectromine^TM^, and SpectroDive^TM^ (Biognosys, Schlieren, DIE, ZH), as well as Mascot Distiller (Matrix Science, Mount Prospect, IL, USA). These software packages accept data from all major MS instruments.

### 4.2. Open-Source Software Alternatives

In addition to commercially available software solutions, several open-source programs and web-based applications have become available for MRM method development and data analysis. One of the most popular options covering the entire experimental workflow is Skyline, which was released by the MacCoss group in 2009 [131]. The software is manufacturer-independent and is designed for the quantification of small molecules, including amino acids and peptides. The software enables the comparison of MS/MS spectra with integrated databases and the development of targeted MRM methods. The functionality of the software is subject to continuous improvement based on user feedback. It includes the prediction of peptide ions, and their transitions based on the uploaded antibody sequence, selected modifications (isotopic labels, PTMs), digestion enzyme and instrument-specific requirements. Furthermore, the software enables the prediction of retention times and facilitates the construction of spectral libraries with the machine learning program Prosit [132]. A range of free tutorials provide entry points for new users to the various aspects of data analysis.

Another freely available and widely used alternative for data evaluation is MaxQuant, developed by groups around Jürgen Cox and Matthias Mann at the Max Planck Institute of Biochemistry [133]. Analogous to Skyline, it is designed to analyze high-throughput mass-spectrometry data in a desktop application. It supports various labeling techniques and algorithms for peak detection, as well as peptide identification with corresponding MS^2^ identification (Andromeda). However, it is more suitable for HRMS data than for MRM data. A substantial collection of published protocols is available for the implementation of MaxQuant in one’s own pipeline for the analysis of mass spectrometric data [134,135].

In addition to the previously discussed options, there are other, more sophisticated software solutions that will be briefly outlined here. OpenMS differs from Skyline in that it provides a flexible programming framework (Python) rather than a ready-to-use desktop interface [136,137]. Functionality includes core algorithms for MS data analysis, such as peak detection, alignment, integration, etc., as well as predefined high-level workflows. MRMPROBS, again, is a stand-alone desktop application aimed at the automatic detection and identification of MRM signals based on probabilistic criteria [138]. Features include XIC panels for efficient quality control (QC) and manual peak correction, but no direct support for peptide targets is provided; target lists must be provided as a text file. MassChroQ is a command-line program supporting both low-resolution (MRM) and HRMS data, as peak quantification is based on XICs rather than feature detection [139]. Peaks from all samples are automatically aligned and exported to a spreadsheet format, allowing integration into data pipelines. MRMAnalyzer similarly focuses on automated pipelines but is implemented as an R package [140,141]. Additionally, MassIVE.quant [142], quantms [143], and MRMPro [144] are examples of recent cloud-based platforms for quantitative proteomics, featuring large-scale online data processing capabilities and extensive QC plots for peak inspection. In the future, artificial intelligence and deep learning algorithms will likely support the user comprehensively by accurately predicting fragmentation patterns and providing “smart” data analysis tools for proteomics in general [145]

## 5. Outlook: Need for Standardized Protocols, Certified Reference Materials, and New Technologies

The use of recombinant antibodies in both therapeutic and diagnostic applications will continue to increase over the next few years. Their exact quantification, both in pure form and in serum, plasma, or blood samples, is therefore essential. As shown in the previous chapters, a variety of peptide-based methods and protocols have been developed in recent decades with different digestion conditions or SIL materials and for each commercial antibody individually (Table 1). It is, therefore, not surprising that different methods can lead to different results, making it difficult for inexperienced users to select a suitable method for the quantification of antibodies. In the authors’ view, standardization of the methods would facilitate more comparable results across research institutions, industry, and clinical studies. In contrast, achieving harmonization regarding the HPLC-MS/MS measurement technology and analysis software used is considered to present a significant challenge.

The need for standardized methods was impressively demonstrated in a study about the quantification of a recombinant human IgG1 SARS-CoV-2 antibody by the Protein Analysis Working Group (PAWG) of the Comité Consultatif pour la Quantité de Matière (CCQM) [106,146]. Participating national metrology institutes (NMIs) showed that there was a large discrepancy in the results (up to 22% difference from reference value) despite the use of isotope dilution mass spectrometry (ID-MS) for peptide-based quantification approaches. Based on this, the digestion conditions and peptide selection for this SARS-CoV-2 antibody were optimized and validated with a reference antibody and AAA as reference method [53]. The authors clearly demonstrated how important it is to use CRMs to develop robust methods and generate accurate results. The need for suitable reference materials and standardized measurements is also emphasized in the clinical field for the quantification of biomarkers, e.g., apolipoprotein A [147,148,149]. Precise quantification is crucial in this context to enable accurate monitoring of biomarkers and effective patient treatment. Furthermore, the use of CRMs makes the results more comparable between different clinical facilities. The implementation of reference materials for quantitative HPLC-MS/MS assay development enables metrological traceability of calibration standards, ensuring that measurement results have a documented and unbroken chain of traceability to SI units [112]. Furthermore, it can also compensate for differences in the device-specific ionizations or software-dependent peak detection and smoothing settings.

A broadly accessible metrological reference material would provide a representative standard to enhance the harmonization of antibody quantification measurements [150]. For the development of standardized methods, the two certified antibody reference materials NISTmAb (RM 8671) and AISTmAb (RM 6208-a) from the National Institute of Standards and Technology (NIST, USA) and National Institute of Advanced Industrial Science and Technology (AIST, Tokyo, Japan) are available [151,152]. Due to the extensive effort needed to establish “certified” values, only a limited number of analytes are certified as reference material. The necessity for CRMs has also become especially evident during the COVID-19 pandemic, particularly in the context of validating analytical tests [103,104,107]. Additionally, the World Health Organization (WHO) also established several antibody reference standards, including the WHO Adalimumab reference standard (ref. 17/236) and the WHO infliximab reference standard (ref. 16/170). However, these standards exhibit inaccuracies in their concentrations [32], highlighting the need for certified, traceable materials that should be characterized through multidisciplinary approaches and evaluated by various laboratories [153]. When selecting suitable calibrators, it is essential to consider the differences in PTMs, as these variations can lead to potential deviations in measurement results [154].

Apart from method standardization and CRM development, new sample preparation technologies may also contribute to faster and more streamlined quantification assay development. We regard innovations in two fields as particularly promising. The first field relates to innovative enzyme technologies for the optimization of protein digestion. A recent method that employs aqueous microdroplet-mediated enzymatic digestion has shown a remarkable decrease in digestion times, achieving results in the millisecond range, in contrast to the several hours typically required by conventional methods [155]. Moreover, this approach has achieved a very high sequence coverage. Similarly, recombinant and immobilized enzyme systems have demonstrated potential in boosting cleavage rates, apparently due to improved stability, inhibition of trypsin self-digestion and higher enzyme-to-protein ratios [59,63]. Furthermore, there are also initial publications on thermostable enzyme variants, for example by inserting mutations or glycosylation [156,157]. We believe that significant further optimization of enzyme systems is feasible due to progress in biotechnology, material science and artificial intelligence (AI)-supported designs. Thereby, the dependency of digestion efficiency on the primary sequence and PTMs of mAb targets could be minimized, resulting in much-improved commutability of the calibrator and SIL materials.

A second field where innovation can have a significant impact on future mAb assays pertains to the production of SIL mAbs. The recently announced mAbXmise kit [95] offers a convenient mixture of several SIL mAbs, enabling the simultaneous quantification of various therapeutic mAbs [32]. The development of further mAb kits may result in more efficient production pipelines, thereby enhancing the broader and more affordable availability of SIL mAbs. This approach could improve compensation for parameters such as digestion conditions and purification from complex matrices, leading to more reproducible results. Ideally, an increase in demand could lead to the commercial availability of SIL mAbs becoming comparable to that of SIL peptides. However, production is expected to remain more complex and costly in the foreseeable future. Cell-free in vitro systems, such as PURE (Protein synthesis Using Recombinant Elements), could provide a cost-effective solution for the production of SIL mAbs [158].

## Figures and Tables

**Table 1:** Top 5 best-selling recombinant antibodies approved for therapeutic application. ^1^ Yearly turnover in 2023 [21]. ^2^ Published peptide-based quantification methods within the last 5 years for the respective mAb and the selected signature peptides.

Generic Name	Antibody Subclass	Year of Approval	Target	Sales ^1^ (Billion USD)	Signature Peptides ^2^
Pembrolizumab	IgG4κ	2021	Programmed cell death protein 1 (PD-1)	25.0	ASGYTFTNYYMYWVR [28] DLPLTFGGGTK [28,29] VTLTTDSSTTTAYMELK [30]
Adalimumab	IgG1κ	2002	Tumor necrosis factor alpha (TNF-α)	14.4	APYTFGQGTK [31,32]
Ustekinumab	IgG1κ	2009	Interleukin (IL)-12 and IL-23	10.9	PGQGYFDFWGQGTLVTVSSSSTK [33] GLDWIGIMSPVDSDIR [29,33]
Daratumumab	IgG1κ	2016	Hydrolase CD38	9.7	SNWPPTFGQGTK [34] LLIYDASNR [35]
Nivolumab	IgG4κ	2014	PD-1	9.0	ASGITFSNSGMHWVR [29,30,36,37]

**Table 2:** Exemplary overview of commercially available MS-grade trypsin and trypsin/Lys-C variants, together with their recommended sample preparation procedures for HPLC-MS/MS analysis and reference to their use for quantification of therapeutic antibodies from Table 1. Products from other manufacturers with similar properties may be equivalent. DTT: Dithiothreitol; TCEP: Tris(2-carboxyethyl)phosphine; IAA: Iodoacetic acid, IAM: Iodoacetamide; opt.: optional.

Product	Special Feature	Denaturation	Reduction	Alkylation	Digestion Conditions	Ref.
**Trypsin**						
Promega GmbH (Walldorf, BW, GER)						
Trypsin Gold/Sequencing Grade	Maximum specificity	8 M Urea 1 h	DTT	IAM 30 min	Overnight 37 °C	[28,31,37]
Trypsin Platinum	Recombinant enzyme, autoproteolytic resistance	8 M GuHCl 30 min	TCEP	IAM 30 min	Overnight 37 °C	
Thermo Fisher Scientific (Waltham, MA, USA)						
Pierce™ Trypsin		1 h at 60 °C or 10 min at 95 °C	DTT	IAA, 30 min	4 to 24 h 37 °C	[29,30]
SMART Digest Trypsin-Kit	Automatable process	-	opt.	opt.	45 min (IgG) 70 °C	
In-Solution Tryptic Digestion and Guanidination Kit	Improved ionization by guanidination of K into homo-R	95 °C 5 min	DTT	IAM, 30 min	2 h at 37 °C or overnight at 30 °C	
Waters Corporation (Milford, MA, USA)						
ProteinWorks eXpress Digest Kit	High throughput of samples possible	Digestion buffer, 80 °C, 10 min	Reduction agent 60 °C, 20 min	Alkylation agent 30 min	2 h 45 °C	[33]
Promise Proteomics (Grenoble, ARA, FRA)						
mAbXmise Kit	Immunocapture cartridges	opt., 4 M to 0.1 M Urea	-	-	30 min to 15 h 37 °C	[32]
**Trypsin/ Lys-C Mix**						
Thermo Fisher Scientific (Waltham, MA, USA)						
EasyPep™ Mini MS Sample Prep Kit	High throughput of samples possible	Lysis solution 95 °C, 10 min	Red. Solution	Alk. Solution	1 to 3 h 37 °C	
Pierce™ Trypsin/ Lys-C Protease Mix		8 M Urea, 1 h at 60 °C or 10 min at 95 °C	DTT	IAM, 30 min	2 to 16 h 37 °C	
Promega GmbH (Walldorf, BW, GER)						
Rapid Digestion– Trypsin/LysC	Fast digestion	-	opt.	opt.	1 h 70 °C	
Trypsin/Lys-C	Quantification	6–8 M Urea, 30 min	DTT	IAM, 30 min	overnight 37 °C	

**Table 3:** Brief overview of frequently used software including latest version for development and evaluation of targeted mAb quantification experiments.^1^ Open-source software. ^2^ Specifically designed for HRMS data.

Manufacturer/Lab	Software
Waters Coporation (Milford, MA, USA)	BioAccord System (UNIFI software, version 1.9.9), TargetLynx™ and QuanOptimize™ (integrated in MassLynx version 4.2)
Thermo Fisher Scientific (Waltham, MA, USA)	Xcalibur (version 4.3), BioPharma Finder^TM^ (version 5.3) and Pinpoint^TM^ (version 4.1)
Agilent Technologies (Santa Clara, CA, USA)	MassHunter (version 12.0)
AB Sciex (Framingham, MA, USA)	Analyst^®^ (version 1.7.3), MultiQuant^TM^ (version 3.0.3) and MRMPilot^TM^ (version 2.1)
Biognosys AG (Schlieren, DIE, ZH)	Spectronaut^TM^ (version 19), Spectromine^TM^ (version 3), and SpectroDive^TM^ (version 12)
MacCoss Lab (Seattle, WA, USA)	Skyline (version 24.1) ^1^
Cox Lab (Martinsried, BY, GER)	MaxQuant (version 2.6.7.0) ^1,2^

## Data Availability

Not applicable.

## Abbreviations

The following abbreviations are used in this manuscript:

AA	Acrylamide
AAA	Amino acid analysis
ABCD	AntiBodies Chemically Defined
ADC	Antibody-drug conjugate
AI	Artificial intelligence
AIST	National Institute of Advanced Industrial Science and Technology
BLAST	Basic Local Alignment Search Tool
CAM	Chloroacetamide
CDR	Complementarity-determining region
C_H_1-3	Constant heavy chains 1 to 3
CHO	Chinese hamster ovary
C_L_	Constant light chain
CRM	Certified reference material
CV	Coefficient of variation
DAR	Drug-antibody ratio
DOC	Sodium dodecyl sulfate
DTT	Dithiothreitol
EMA	European Medicines Agency
ESI	Electrospray ionization
F_AB_	Antigen-binding sites of antibody
F_C_	Fragment crystallizable region
FDA	Food and Drug Administration
HAMA	Human anti-mouse antibody
HILIC	Hydrophilic interaction chromatography
HPLC-MS/MS	High-performance liquid chromatography-tandem mass spectrometry
HRMS	High-resolution mass spectrometry
IAA	Iodoacetic acid
IAC	Immunoaffinity capture
IAM	Iodoacetamide
ID-MS	Isotope dilution mass spectrometry
IEX	Ion exchange
IgG1	Immunoglobulin G antibody class 1
IgG4	Immunoglobulin G antibody class 4
IL-12	Interleukin-12
IL-23	Interleukin-23
IMGT	ImmunoGenetics Information system
K	Lysine
LBA	Ligand binding assay
LOD	Limit of detection
LOQ	Limit of quantification
mAb	Therapeutic monoclonal antibody
MAM	Multi-attribute method
MRM	Multiple reaction monitoring
MS	Mass spectrometry
NIST	National Institute of Standards and Technology
NMI	National Metrology Institute
opt.	Optional
PD-1	Programmed cell death protein 1
PSAQ	Protein standard absolute quantification
PTM	Post-translational modification
PURE	Protein synthesis Using Recombinant Elements
QC	Quality control
QqQ	Triple-quadrupole
Q-Trap	Quadrupole-ion trap
R	Arginine
RP	Reversed phase
RSD	Relative standard deviation
SDS	Sodium dodecyl sulfate
SI units	International standard system of units
SIL	Stable isotope-labeled
SPE	Solid-phase extraction
TCEP	Tris(2-carboxyethyl)phosphine
tMRM	Triggered MRM
TNF-α	Tumor necrosis factor-alpha
TPCK	Tosyl-phenylalanyl-chloromethyl-ketone
V_H_	Variable heavy chain
V_L_	Variable light chain
WHO	World Health Organization
XIC	Extracted ion chromatogram
